# Calpain-dependent disruption of nucleo-cytoplasmic transport in ALS motor neurons

**DOI:** 10.1038/srep39994

**Published:** 2017-01-03

**Authors:** Takenari Yamashita, Hitoshi Aizawa, Sayaka Teramoto, Megumi Akamatsu, Shin Kwak

**Affiliations:** 1 Graduate School of Medicine, The University of Tokyo, 7-3-1 Hongo, Bunkyo-ku, Tokyo 113-0033, Japan; 2 Department of Neurology, Tokyo Medical University, 6-7-1 Nishishinjuku, Shinjuku-ku, Tokyo 160-0023, Japan; 3 Clinical Research Center for Medicine, International University of Health and Welfare, 6-1-14 Konodai, Ichikawa, Chiba 272-0827, Japan

## Abstract

Nuclear dysfunction in motor neurons has been hypothesized to be a principal cause of amyotrophic lateral sclerosis (ALS) pathogenesis. Here, we investigated the mechanism by which the nuclear pore complex (NPC) is disrupted in dying motor neurons in a mechanistic ALS mouse model (adenosine deaminase acting on RNA 2 (ADAR2) conditional knockout (AR2) mice) and in ALS patients. We showed that nucleoporins (Nups) that constituted the NPC were cleaved by activated calpain via a Ca^2+^-permeable AMPA receptor-mediated mechanism in dying motor neurons lacking ADAR2 expression in AR2 mice. In these neurons, nucleo-cytoplasmic transport was disrupted, and the level of the transcript elongation enzyme RNA polymerase II phosphorylated at Ser2 was significantly decreased. Analogous changes were observed in motor neurons lacking ADAR2 immunoreactivity in sporadic ALS patients. Therefore, calpain-dependent NPC disruption may participate in ALS pathogenesis, and inhibiting Ca^2+^-mediated cell death signals may be a therapeutic strategy for ALS.

Amyotrophic lateral sclerosis (ALS) is the most common adult-onset motor neuron disease of unknown etiology. It has long been known that both nuclear volume and RNA contents of motor neurons are decreased in ALS patients compared to healthy control subjects^1^. Recent findings of ALS-related genes encoding RNA-binding proteins (RBPs) such as TDP-43 and FUS and of a reduction or loss of these RBPs from the nuclei of anterior horn cells (AHCs) in ALS patients suggest a role of RNA dysregulation in ALS pathogenesis^2^,^3^,^4^. RNAs and RBPs are transported between the nucleus and the cytoplasm, and the nuclear pore complex (NPC) functions as a gateway for nucleo-cytoplasmic transport of these molecules^5^,^6^. Disruption of nucleo-cytoplasmic transport or the dysfunction of the NPC is a predicted mechanism underlying cell death^7^,^8^,^9^,^10^; and its potential role in ALS pathogenesis has also been suspected. Indeed, the gene encoding the GLE1 protein, a nucleoporin (Nup) that is a constituent of the NPC, has been associated with ALS^11^, and morphological changes in the nuclear membrane upon disruption of Nups have been observed in motor neurons of patients with sporadic ALS or with SOD1-associated ALS^12^,^13^. Moreover, nucleo-cytoplasmic transport through the NPC was found to be disrupted in cultured cells and in animals expressing the ALS-associated *C9orf72* gene harboring an expanded GGGGCC (G_4_C_2_) hexanucleotide repeat sequence^14^,^15^,^16^. However, the manner in which NPC is disrupted in ALS motor neurons remains unclear.

Adenosine deaminase acting on RNA (ADAR)2 is a member of the ADAR family, which catalyzes the adenosine-to-inosine (A-to-I) conversion in pre-mRNA. Progressive down-regulation of ADAR2 with resultant failure of the A-to-I conversion at the glutamine/arginine (Q/R) site of GluA2, the Ca^2+^-regulating subunit of AMPA receptors, has been demonstrated in motor neurons of most patients with sporadic ALS^17^,^18^,^19^. Furthermore, conditional ADAR2 knockout mice (AR2 mice) displayed the ALS phenotype resulting from progressive degeneration of motor neurons, and also exhibited TDP-43 mislocalization that resembled TDP-43 pathology^20^,^21^, the most reliable pathological hallmark of ALS. This behaviorally and pathologically ALS-like phenotype of AR2 mice results from excess influx of Ca^2+^ through AMPA receptor complexes containing Q/R site-unedited GluA2 subunits^20^,^21^; continuous Ca^2+^ influx through these abnormal AMPA receptors activates the Ca^2+^-dependent cysteine protease calpain, which cleaves TDP-43 into aggregation-prone fragments that serve as seeds for TDP-43 pathology^20^,^21^,^22^. The above evidence indicates that the ADAR2-deficient motor neurons in AR2 mice mimic the pathogenetic mechanism of sporadic ALS. Notably, the motor neurons of AR2 mice possess abnormal vacuoles during the course of death, and these nuclear vacuoles disappear when Ca^2+^ influx through AMPA receptors is normalized^23^. These pieces of evidence suggest the involvement of Ca^2+^-dependent dysregulation of nucleo-cytoplasmic transport in the slow death of motor neurons in AR2 mice and ALS patients. In this study, we investigated how expression of Ca^2+^-permeable AMPA receptors disrupts the nucleo-cytoplasmic transport machinery in AR2 mice and whether similar alterations are present in motor neurons of ALS patients.

## Results

### Calpain-dependent degradation/denaturation of the NPC

**Figure 1:**
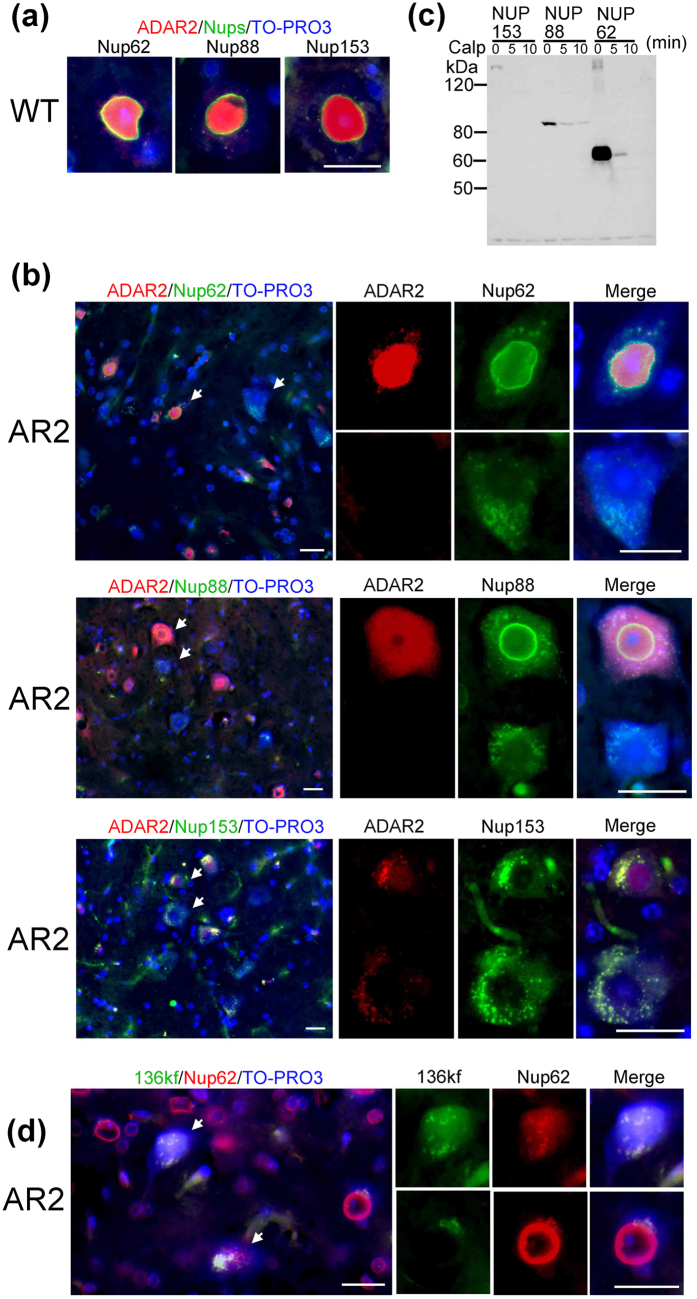
The NPC was degraded and denatured by calpain. (**a,b**) Representative immunofluorescence staining for ADAR2 and Nups in AHCs of a wild-type (**a**) and an AR2 mouse (**b**). Immunofluorescence assays showed that the Nups (green) were localized to the perinuclear region of ADAR2-postive AHCs but lacked detectable expression in ADAR2-negative AHCs of an AR2 mouse. Arrows indicate AHCs that appear in the magnified images. (**c**) Western blotting for Flag. Flag-Nup153, Flag-Nup88, and Flag-Nup62 were cleaved by recombinant calpain-I. (**d**) AHCs with high immunoreactivity for the 136 kDa fragment of alpha-spectrin (136 kf) in an AR2 mouse did not exhibit normal perinuclear Nup62 immunoreactivity (arrow). TO-PRO-3 is a cellular marker (blue). Scale bar, 20 μm.

**Figure 2:**
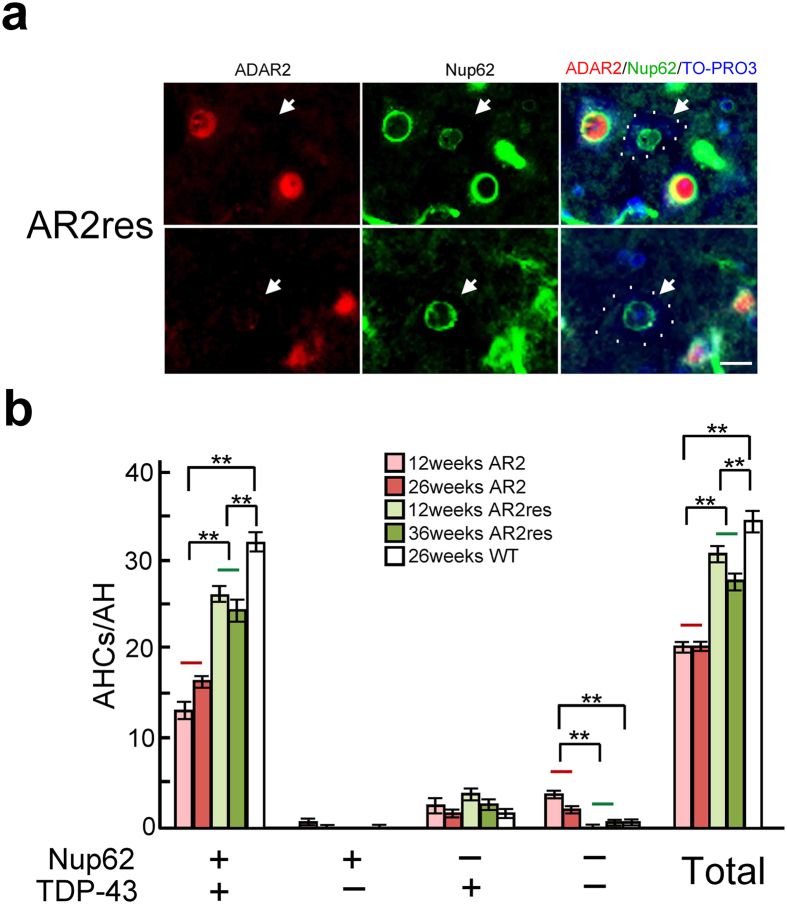
Rescue of Nup62 in AHCs of AR2res mouse. (**a**) Irrespective of ADAR2 expression (arrow), all AHCs in an AR2res mouse expressed Nup62 protein in the perinuclear region. TO-PRO-3 is a cellular staining marker. Scale bars, 20 μm. (**b**) The numbers of AHCs that were notably immunoreactive for Nup62 and TDP-43 in the AR2, AR2res and wild-type (WT) mice at different ages. The mean number of AHCs ± s.e.m. in a single anterior horn is indicated (n = 4–5, **P < 0.005 compared to the levels in wild-type or AR2 mice, Mann-Whitney U-test). +: positive immunoreactivity; −: negative immunoreactivity.

First, we investigated whether the NPC was disrupted in ADAR2-deficient motor neurons in AR2 mice. Nup immunoreactivity was observed in the nuclear membrane and the perinuclear region of AHCs in wild-type mice (Fig. 1a and Supplementary Fig. S1) and ADAR2-positive AHCs in AR2 mice (Fig. 1b). In contrast, immunoreactivity for the Nup62, Nup88, and Nup153 proteins was absent from the perinuclear region but was detected as irregular granule-like fluorescence in the cytoplasm in ADAR2-deficient AHCs in AR2 mice (Fig. 1b). Because the Ca^2+^-dependent protease calpain is abnormally activated in AHCs lacking ADAR2 expression in AR2 mice^21^, we next examined whether calpain cleaves Nups. An *in vitro* calpain cleavage assay demonstrated that Flag-Nups were effectively cleaved by calpain (Fig. 1c), which suggested that the lack of Nup immunoreactivity in ADAR2-deficient AHCs likely resulted from cleavage of Nups by activated calpain. To investigate calpain activity in ADAR2-deficient AHCs, we used polyclonal antibodies against the 136 kDa fragment of alpha-spectrin (136kf), a calpain-dependent fragment of alpha-spectrin that is a marker of prominent activation of calpain^24^. The ADAR2-deficient AHCs, but not ADAR2-positive AHCs, exhibited 136kf signals in the nuclei that were devoid of normal ring-like staining for Nup62 (Fig. 1d and Supplementary Fig. S2). Hence, to assess whether the lack of Nup62 in AR2 mouse AHCs was induced by the activation of calpain in response to an amplified Ca^2+^ influx through the AMPA receptors, we examined the changes in Nup62 and ADAR2 levels in AR2res (AR2/GluR-B^R/R^) mice, in which the endogenous GluA2 alleles were replaced with the GluR-B^R^ allele, which encodes Q/R site-edited GluA2^20^,^25^,^26^. The motor neurons in AR2res mice express basally Ca^2+^-impermeable AMPA receptor complexes containing edited GluA2 subunits even in the absence of ADAR2. The ADAR2-deficient AHCs displayed normal Nup62 immunoreactivity in the AR2res mice at 3 and 9 months of age (Fig. 2a, arrows). Because TDP-43 was cleaved by calpain and exhibited mislocalization and motor neuron death was induced in a Ca^2+^-dependent manner in the ADAR2-deficient motor neurons of AR2 mice^21^,^22^,^27^,^28^ (Supplementary Fig. S3), we compared the immunoreactivity to Nup62 and TDP-43 in motor neurons between the AR2 and AR2res mice. The numbers of AHCs positive for both Nup62 and TDP-43 were higher in the AR2res mice than in the AR2 mice, and virtually no motor neurons in the AR2res mice were negative for both Nup62 and TDP-43 (Fig. 2b). Moreover, the number of choline acetytrasferase (ChAT)-positive AHCs that were Nup62-positive was decreased in the AR2 mice compared to that in the wild type mice (Supplementary Fig. S3). These results indicate that activated calpain degraded and denatured the NPC via up-regulation of Ca^2+^-permeable AMPA receptors in motor neurons of AR2 mice in a manner dependent on ADAR2 ablation.

### Defective nucleo-cytoplasmic transport in ADAR2-deficient motor neurons

**Figure 3:**
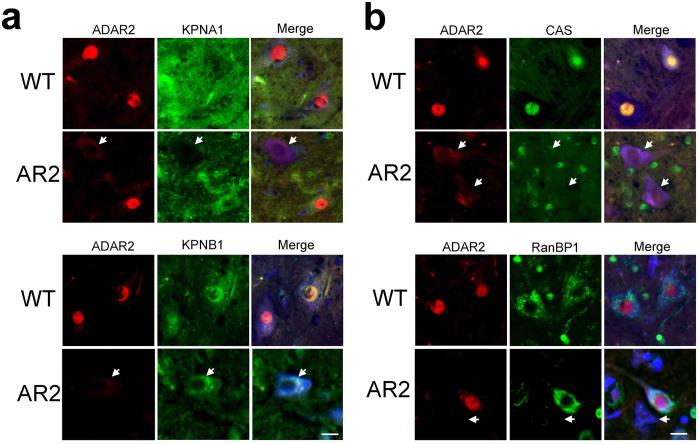
ADAR2 down-regulation causes nucleo-cytoplasmic transport defects in the motor neurons of AR2 mice. Immunofluorescence staining for ADAR2 (red) and for proteins involved in nucleo-cytoplasmic transport (green) in mouse AHCs. (**a**) The ADAR2-deficient AHCs in an AR2 mouse (arrow) lacked normal nuclear KPNA1 and KPNB1 immunoreactivity. (**b**) The ADAR2-deficient AHCs lacked CAS immunoreactivity, and exhibited depleted RanBP1 immunoreactivity (arrow). TO-PRO-3 is a cellular marker (blue). Scale bar, 20 μm.

We next examined the changes in the subcellular localization of proteins associated with classical nuclear import and the importin recycling system. The cargo proteins karyopherin, importin, exportin, and transportin play important roles in nucleo-cytoplasmic transport, and the recycling of nucleo-cytoplasmic transporters is regulated by Ran GTPases such as RanGAP and RanBP1^29^. We found that the subcellular localization of KPNA1 and KPNB1, members of the karyopherin protein family, was different between ADAR2-positive and ADAR2-deficient AHCs. ADAR2-positive AHCs in wild-type and AR2 mice exhibited KPNA1 immunoreactivity in the cytoplasm and KPNB1 immunoreactivity in the nucleus and the cytoplasm. Alternatively, ADAR2-deficient AHCs in AR2 mice were devoid of specific KPNA1 and KPNB1 immunoreactivity (Fig. 3a). Immunoreactivity for cellular apoptosis susceptibility protein (CAS), also known as exportin-2, was predominant in the nucleus of ADAR2-positive motor neurons but was faintly detected in the cytoplasm of ADAR2-deficient motor neurons. Immunoreactivity for RanBP1, which controls assembly/disassembly of certain karyopherin-cargo complexes^30^, was predominantly cytoplasmic in ADAR2-positive motor neurons but was nuclear or undetectable in ADAR2-deficient motor neurons (Fig. 3b and Supplementary Fig. S4).

**Figure 4:**
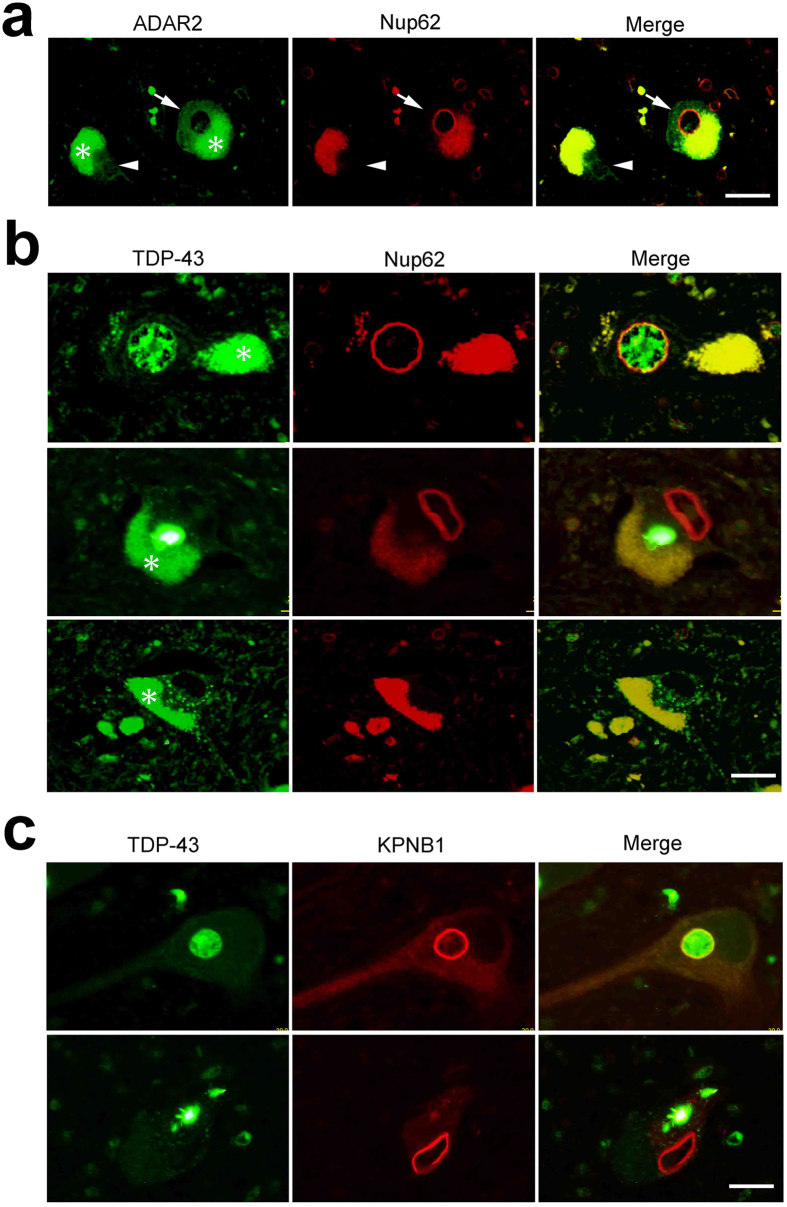
Nucleo-cytoplasmic transport defects in ADAR2-deficient motor neurons of sporadic ALS patients. (**a**) An ADAR2-positive AHC expressed Nup62 in the perinuclear region (arrow), whereas an ADAR2-negative AHC lacked Nup62 protein expression (arrowhead). (**b**) A TDP-43-positive AHC in a sporadic ALS patient expressed the Nup62 protein in a round perinuclear region, whereas an AHC with cytoplasmic TDP-43 inclusions exhibited distorted perinuclear Nup62 immunoreactivity and a TDP-43-negative AHC lacked Nup62 protein expression. (**c**) A TDP-43-positive AHC expressed the KPNB1 protein in the perinuclear region and the nucleus, whereas AHCs with TDP-43 inclusions showed irregular and discontinuous nuclear KPNB1 protein immunoreactivity. Asterisks in all panels indicate autofluorescence of lipofuscin. Scale bars, 30 μm.

Because TDP-43 pathology in motor neurons is a pathological hallmark of ALS^31^,^32^ and because motor neurons exhibiting TDP-43 pathology invariably lack ADAR2 immunoreactivity^33^, we next examined Nup62 and KPNB1 immunoreactivity in combination with ADAR2 or TDP-43 immunoreactivity in motor neurons of sporadic ALS patients. ADAR2-positive neurons, but not ADAR2-deficient neurons, showed normal ring-like Nup62 immunoreactivity in the perinuclear region, (Fig. 4a). AHCs with nuclear TDP-43 immunoreactivity exhibited normal perinuclear Nup62 immunoreactivity (Fig. 4b upper panels), whereas AHCs with TDP-43-positive cytoplasmic inclusions exhibited Nup62 immunoreactivity along the tortuous nuclear membrane or no Nup62 immunoreactivity (Fig. 4b middle and lower panels). Similarly, in contrast to the round perinuclear KPNB1 immunoreactivity in motor neurons with nuclear TDP-43 (Fig. 4c upper panels), motor neurons with TDP-43-positive inclusions exhibited no KPNB1 immunoreactivity or irregular and discontinuous perinuclear KPNB1 immunoreactivity (Fig. 4c lower panels). Moreover, the KPNA1 and KPNB1 immunostaining in the ADAR2-deficient AHCs of AR2res mice exhibited normal distribution (Supplementary Fig. S5). Because ADAR2-lacking AHCs in the AR2res mice express edited GluA2 and hence Ca^2+^-impermeable AMPA receptors, these results indicate that the NPC and the nucleo-cytoplasmic transport system are altered in ADAR2-deficient AHCs of sporadic ALS patients in a manner dependent on Ca^2+^ influx.

### Altered gene expression in motor neurons with disrupted NPCs

**Figure 5:**
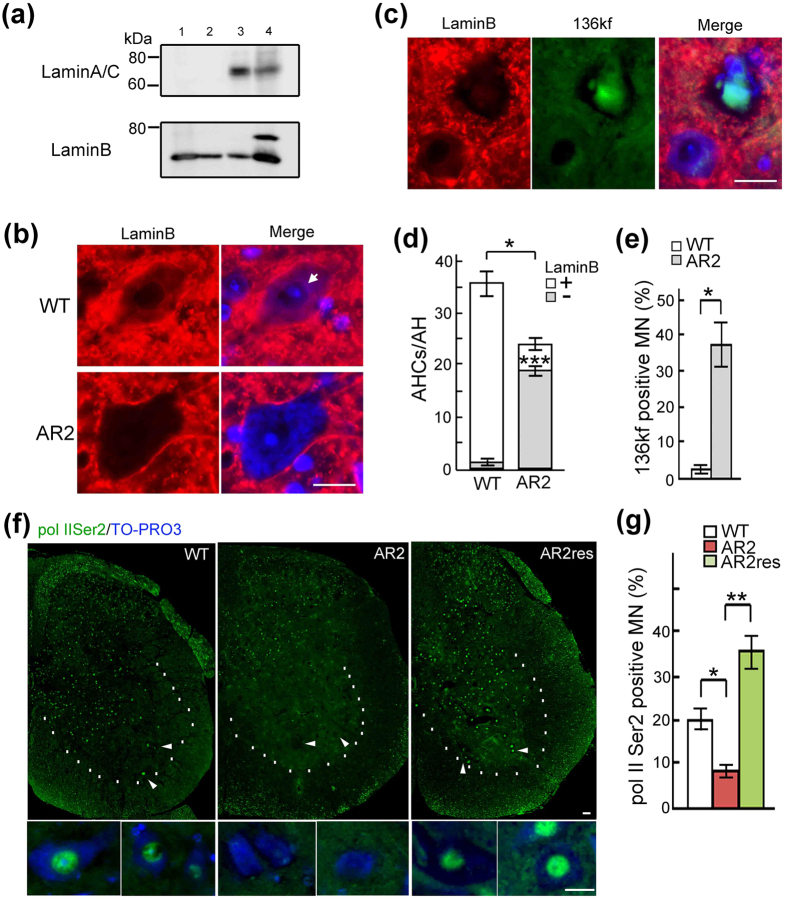
Nuclear dysfunction in motor neurons with NPC disruption. (**a**) Western blot showed that lamin B is the predominant member of the lamin family in the mouse brain and spinal cord. The lanes indicated (lane 1: brain, lane 2: spinal cord, lane 3: muscle, lane 4: liver). (**b**) Cytoplasmic and perinuclear lamin B immunoreactivity was detected in a wild-type mouse AHC but not in an AR2 mouse AHC. Scale bar, 20 μm. (**c**) Immunoreactivity for 136kf was observed in AR2 mouse AHCs deficient lamin B expression. Scale bar, 20 μm. (**d**) The number of lamin B-positive AHCs. Means (columns) and s.e.m. (bars) are indicated (The number of total AHCs; n = 5; *P = 0.0119 compared to the wild-type levels. The number of lamin B-negative AHCs; n = 12; ***P < 0.001 compared to the wild-type levels, Student’s t-test). (**e**) The proportion of AHCs positive for 136 kf among all AHCs in AR2 mice and wild-type mice, both at 28 weeks of age. Means (columns) and s.e.m. (bars) are indicated (n = 4; *P = 0.0284 compared to the wild-type levels, Student’s t-test). (**f,g**) The proportion of AHCs positive for pol II Ser2 (green) among the AHCs in AR2, wild-type or AR2res mice (28 weeks of age). Significantly fewer pol II Ser2-positive neurons were detected in AR2 mice than in wild-type and AR2res mice. TO-PRO-3 is a cellular marker (blue). Means (columns) and s.e.m. (bars) are indicated (n = 5; *P = 0.0105 and **P < 0.001 compared to the wild-type or AR2res levels, Student’s t-test).

The observation of tortuous ring-like Nup62 and KPNB1 immunoreactivity (Fig. 4) suggested that the nuclear membranes of ADAR2-deficient motor neurons in ALS patients were distorted. Members of the lamin protein family are nuclear envelope proteins that are crucial for not only maintaining nuclear structure but also regulating transcription by modulating chromatin organization^34^,^35^,^36^. Lamin B, but not lamin A/C, was expressed in the mouse brain and spinal cord (Fig. 5a), and lamin B was predominantly localized to the cytoplasm, particularly the perinuclear region, in motor neurons of wild-type mice (Fig. 5b,d). In contrast, lamin B expression was not detected in ADAR2-deficient AHCs of AR2 mice (Fig. 5b,d). These AHCs devoid of both lamin B and ADAR2 expression exhibited 136kf expression in the nucleus (Fig. 5b,c,d,e and Supplementary Fig. S6).

Next, we tested whether these structural and functional changes in the nucleo-cytoplasmic transport system affected gene expression. The expression of RNA polymerase II phosphorylated at Ser2 (pol II Ser2), a transcript elongation enzyme, was markedly decreased in AHCs of AR2 mice (Fig. 5f). In addition, the number of pol II Ser2-positive AHCs was significantly lower in AR2 mice than in wild-type mice (Fig. 5g and Supplementary Fig. S7). Moreover, these changes in AR2 mice were rescued in AR2res mice (Fig. 5f,g and Supplementary Fig. S7).

## Discussion

In this study, we showed that physiological nucleo-cytoplasmic transport was disrupted in dying ADAR2-deficient motor neurons via a Ca^2+^-permeable AMPA receptor-mediated mechanism in AR2 mice, a mechanistic mouse model of sporadic ALS. In this model, Nups, the components of the NPC, were cleaved by the Ca^2+^-dependent cysteine protease calpain, and immunoreactivity for Nups and cargo proteins involved in nucleo-cytoplasmic transport was markedly altered in ADAR2-deficient AHCs. Notably, analogous changes in the expression of Nups and importins were observed in the spinal AHCs of patients with ALS in a manner dependent on ADAR2 down-regulation.

The NPC serves as a gateway for nucleo-cytoplasmic transport, and karyopherins (KPNA1 and KPNB1), CAS and RanBP1 are cargo proteins involved in nuclear import, nuclear export and recycling of nucleo-cytoplasmic transporters, respectively^29^. Export of RanBP1 from the nucleus to the cytoplasm depends on the chromosome region maintenance 1 (CRM1)-dependent nuclear export system^30^, and the down-regulation of RanBP1 resulted in the death of cultured cells^37^,^38^. Therefore, the finding of marked alteration of the subcellular localization of nucleo-cytoplasmic cargo proteins in this study suggests robust impairment of nuclear function due to cleavage of NPC components such as Nups by calpain in ADAR2-deficient AHCs.

Malfunction of nucleo-cytoplasmic transport caused by Ca^2+^ dysregulation has been proposed to occur in degenerative diseases^39^, and excess Ca^2+^ influx reportedly disrupts nucleo-cytoplasmic transport as a result of calpain-dependent degradation of NPC components^10^,^40^,^41^, including nuclear lamins and Nups. Disruption of the NPC enabled redistribution of calpains from the cytoplasm to the nucleus across the nuclear membrane during an acute excitotoxic insult^10^, but the pathogenic role of NPC disruption and the occurrence of NPC disruption during the slow neuronal death process have not been demonstrated. The present results indicated that activated calpain was localized to the nucleus, suggesting that nuclear dislocation of calpain occurred not only upon acute excitotoxic insult but also during a non-acute process of neuronal death. Ca^2+^ freely crosses nuclear membrane, and changes in the nucleoplasmic Ca^2+^ levels can directly regulate gene expression^42^. However, calpain becomes mislocalized in the nucleus only after disruption of nucleo-cytoplasmic transport. The loss of expression of the structural nuclear envelope protein lamin B in ADAR2-deficient AHCs of AR2 mice is likely due to its cleavage by mislocalized calpain. GLE1, the product of the recently identified ALS-associated gene *GLE1*^11^, was also found to be cleaved by calpain1 *in vitro* (data not shown). Calpain-1 mislocalized to the nucleus cleaved histone H3, thereby affecting gene expression to induce epithelial cell death during mammary gland differentiation^41^. Disrupted nucleo-cytoplasmic transport of RNAs and RBPs including RanBP1 may accelerate cell death signaling, and down-regulation of pol II Ser2 as observed in this study likely reflects overall dysregulation of gene expression in dying motor neurons in AR2 mice.

Disrupted nucleo-cytoplasmic transport has recently attracted interest as a potential cause of ALS^14^,^15^,^16^,^43^ in association with the leading hypothesis of dysregulation of RNA metabolism^2^, but the mechanism underlying ALS pathology remains largely unresolved. In this study, we demonstrated that degeneration of the NPC in motor neurons of ALS patients was associated with ADAR2 down-regulation and TDP-43 pathology, which suggests the presence of an underlying calpain-dependent mechanism, as noted in AR2 mice. Progressive down-regulation of ADAR2 is a characteristic feature of motor neurons in sporadic ALS patients^25^, which leads to dysregulation of RNA metabolism due to failure of the A-to-I conversion in pre-mRNA. The crucial function of ADAR2 is RNA editing at the GluA2 Q/R site, but the main mediators of the downstream events leading to cell death are Ca^2+^ influx and activity of calpains, which degrade physiologically functioning molecules, including TDP-43 and Nups, rather than failure of the A-to-I conversion in RNAs^20^,^21^. Interestingly, present results suggest that dysregulation of RNA metabolism by ADAR2 down-regulation indirectly affects gene expression via altering physiologically crucial protein functions.

ADAR2-deficient motor neurons in both AR2 mice and sporadic ALS patients share several characteristics, including slow progressive death of motor neurons except for those innervating extraocular muscles, expression of Q/R site-unedited GluA2, and mislocalization of TDP-43^17^,^18^,^19^,^20^,^21^,^33^. Here, we observed calpain-dependent degradation of Nups and nucleo-cytoplasmic transport elements in dying motor neurons as an additional common feature between AR2 mice and those of sporadic ALS patients. The multifaceted pathological similarities between sporadic ALS patients and AR2 mice lend further support to the hypothesis that ADAR2 down-regulation plays a pivotal role in ALS pathogenesis. The present results suggest that the role of disruption of nucleo-cytoplasmic transport and the resultant dysregulation of gene expression in calpain-mediated cell death^44^,^45^ is involved in the mechanism underlying the death of motor neurons in ALS patients. Future studies will be required to elucidate the molecular cascade whereby disrupted nucleo-cytoplasmic transport causes cell death, which could aid in the development of novel mechanism-based therapies for FTD/ALS.

## Methods

### Antibodies

The following primary antibodies were used: rabbit anti-FLAG (Cell Signaling Technology); mouse anti-nucleoporin p62 (BD Transduction Labs); mouse anti-human Nup88 (BD Transduction Labs); mouse anti-Nup153 (QE5, Abcam); rabbit anti-136kf (specific calpain-dependent cleavage fragment of alpha-spectrin, a gift from Dr. T.C. Saido, Laboratory for Proteolytic Neuroscience, RIKEN Brain Science Institute^24^); rabbit anti-RNA polymerase II CTD repeat YSPTSPS (phospho-S2) (Abcam); mouse anti-RNA polymerase II H5 (Covance); goat anti-lamin B (Santa Cruz Biotechnologies, Inc.); mouse anti-lamin A/C (Santa Cruz Biotechnologies, Inc.); rabbit anti-nucleoporin p62 (Abcam); rabbit anti-Nup88 (Abcam); rabbit anti-human beta-actin (IMGENEX Corp.); sheep anti-rat RED1 (ADAR2, Exalpha Biologicals, Inc.); rabbit anti-ADARB1 (ADAR2, Sigma Aldrich); rabbit anti-KPNA3 (Abcam); rabbit anti-KPNA1/SRP1 (Abcam); rabbit anti-CAS (Abcam); mouse anti-transportin 1 (Abcam); rabbit anti-CRM1 (Santa Cruz Biotechnologies, Inc.); rabbit anti-karyopherin beta 3 (Santa Cruz Biotechnologies, Inc.); rabbit anti-karyopherin beta 1 (Santa Cruz Biotechnologies, Inc.); rabbit anti-KPNA2 (Abcam); and rabbit anti-RanBP1 (Novus Biotech, Inc., Littleton, CO, USA); goat anti-ChAT (Milllipore).

The following secondary antibodies were used for immunohistochemistry: Alexa Fluor 488 donkey anti-sheep IgG, Alexa Fluor 488 goat anti-mouse IgG, Alexa Fluor 488 donkey anti-goat IgG, Alexa Fluor 647 chicken anti-rabbit IgG, Alexa Fluor 647 chicken anti-mouse IgG (Invitrogen). Peroxidase-conjugated goat anti-rabbit IgG and peroxidase-conjugated horse anti-mouse IgG (Cell Signaling Technology, Inc., Danvers, MA, USA) were the secondary antibodies used for immunoblot analyses.

### Construction of plasmids

To generate full-length human Nup62, Nup88 and Nup153 expression constructs, we first amplified the Nup62, Nup88 and Nup153 coding regions from cDNA generated from human spinal cord RNA using the following primers: hNUP62-XhoIup1 (5′-AAAAACTCGAGATGAGCGGGTTTAATTTTGGAGGCACTG-3′) and hNUP62-SalIdown1 (5′-AAAAAGTCGACTCAGTCAAAGGTGATCCGGAAGCTGCGCTC-3′); hNUP88-KpnIup1 (5′-AAAAAGGTACCATGGCGGCCGCCGAGGGACCGGTGGGCGAC-3′) and hNUP88-SalIdown1 (5′-AAAAAGTCGACTCAGAAGTTTACATGATTGCGGATATCATTG-3′); or hNUP153-KpnIup1 (5′-AAAAAGGTACCATGGCCTCGGGAGCCGGAGGAGTCGGAG-3′) and hNUP153-SalIdown1 (5′-AAAAAGTCGACTTATTTCCTGCGTCTAACAGCAGTCTTTATC-3′). After gel purification, the PCR products were digested with *SalI* and either *XhoI* or *KpnI,* and the appropriate fragments were cloned into a pCI mammalian expression vector (Promega) that was digested with the same restriction enzymes. Flag-tagged full-length Nup62, Nup88 and Nup153 constructs were generated using a KOD Plus mutagenesis kit (TOYOBO). All constructs were verified by DNA sequencing.

### Cell culture and transfection

HeLa cells were cultured in MEM-alpha medium (WAKO, Tokyo, Japan) supplemented with 10% fetal bovine serum (Invitrogen, Carlsbad, CA, USA), 100 U/ml penicillin and 100 μg/ml streptomycin (Invitrogen) in 5% CO_2_ at 37 °C. The cells were seeded in 6-well plates at a density of 3.5 × 10^4^ cells/cm^2^. The HeLa cells were transfected with 2.5 μg of an expression plasmid using Lipofectamine 3000 transfection reagent (Invitrogen). The cells were cultured for 72 hours after transfection and were then harvested.

### *In vitro* calpain and caspase assays

HeLa cells were homogenised by sonication in 10 volumes of extraction buffer (50 mM HEPES, pH 7.5, 1 mM EDTA, 100 mM NaCl, 10 mM DTT and 0.1% CHAPS). The homogenate was centrifuged at 1,000 g for 10 min at 4 °C. The supernatant was incubated at 30 °C with 3.0 units of purified human erythrocyte μ-calpain (calpain-I) in extraction buffer with 5 mM CaCl_2_. The reaction was stopped by addition of SDS sample buffer containing 62.5 mM Tris-HCl, pH 6.8, 4% SDS, 10% glycerol, 50 mM DTT, and 0.1% bromophenol blue. The samples were boiled at 100 °C for 5 min.

### Animals

The mutant animals used in this study were homozygous (*ADAR2*^*flox/flox*^/VAChT-Cre.Fast) conditional ADAR2 knockout (AR2) mice. In AR2 mice, the ADAR2 gene is conditionally ablated in motor neurons using VAChT promoter-driven Cre in the Cre/loxP system^20^. AR2res (*ADAR2*^*flox/flox*^/VAChT-Cre.Fast/GluR-B^*R/R*^) mice were generated by crossing AR2 mice with GluR-*B*^*R/R*^ mice, in which the endogenous GluA2 gene was engineered to encode directly edited GluA2. In the AR2res mice, ADAR2-deficient motor neurons did not undergo neuronal death by 6 months of age^20^. Both genders of AR2 and AR2res mice were used in this study. C57BL/6 J mice (Oriental Yeast Co., Ltd.) of the same age were used as the WT control of the same strain. In this study, homozygous AR2 mice at 12, 26, 28, 36, 38 weeks of age and AR2res mice at 12, 28, 36 weeks of age and wild type mice at 26, 28, 36 weeks of age were used for all experiments. The mice were housed at two to three per cage on a 12 h: 12 h light-dark cycle with free access to food and water. All of the studies were approved by the Committee on Animal Handling at the University of Tokyo and were performed in accordance with the guidelines for animal experiments of the Ministry of Education, Culture, Sports, Science and Technology, Japan.

### Human material and preparation of samples

Human spinal cord specimens were fixed in 10% neutral buffered formalin for approximately 7 days and then embedded in paraffin. Serial 7-μm sections were sliced. The sections were mounted on slides, deparaffinized in xylene, hydrated with a graded ethanol series and heated at 120 °C for 2 min for antigen retrieval. The sections were then washed with phosphate-buffered saline (PBS) and incubated with a primary antibody overnight at 4 °C. Written informed consent for autopsy and approval for the use of autopsy tissue specimens for research purposes was obtained before death from all subjects, and the research project has been approved by the Institutional Human Ethics Committees of Tokyo Medical University. All methods were performed in accordance with the relevant guidelines and regulations by including a statement in the methods section to this effect.

### Protein extraction

The samples, including frozen mouse muscles, livers, brains and spinal cords, were homogenized in 10 volumes of extraction buffer (5 mM cysteine and 150 mM imidazole, pH 7.5) containing a protease inhibitor cocktail supplemented with 5 mM ethylenediaminetetraacetic acid (EDTA) (Pierce). After the homogenate was centrifuged at 1,000 × g for 10 min at 4 °C, SDS sample buffer (62.5 mM Tris-HCl, pH 6.8, 4% SDS, 10% glycerol, 50 mM DTT, and 0.1% bromophenol blue) was added, and the sample was boiled at 100 °C for 5 min immediately prior to SDS-PAGE analysis.

### Western blot analysis

Each sample was subjected to 7.5%, 12.5% or 15% (v/v) SDS-PAGE using a Tris-glycine buffer system, and the separated proteins were transferred to polyvinylidene difluoride membranes (Millipore). The membranes were blocked with 3% (v/v) skim milk and 1% (v/v) bovine serum albumin (BSA) in PBS containing 0.1% (w/v) Tween-20 for 3 hours, followed by incubation at 4 °C overnight with the indicated primary antibody at an appropriate dilution (1:2,000) in PBS containing 3% (v/v) skim milk, 1% (v/v) BSA, and 0.1% (w/v) Tween-20. The membranes were washed and then incubated with a horseradish peroxidase-conjugated secondary antibody (1:2,000) (Cell Signaling Technology, Inc., Danvers, MA, USA) at room temperature for 1 hour. Signals were detected using Pierce Western Blotting Substrate Plus (Thermo Scientific, Southfield, MI, USA). The intensities of specific bands were quantitatively measured using an LAS-3000 mini imaging system (FujiFilm Co., Tokyo, Japan) equipped with Multi-Gauge Ver3.0 software (FujiFilm). As an internal control, the expression of β-actin was measured.

### Immunofluorescence analysis

Under deep anesthesia with isoflurane, mice were transcardially perfused with 3.5% paraformaldehyde and 0.5% glutaraldehyde in PBS. The spinal cords were removed and immersed in serially increasing concentrations of sucrose in PBS (final sucrose concentration of 30%). The sucrose-immersed spinal cord specimens were sliced to a thickness of 10 μm using a cryostat (Model LEICA CM1850; LEICA). The sections were blocked with 10% (v/v) skim milk in PBS. Immunofluorescence staining of the sections was performed using the indicated primary antibodies. The sections were then incubated with Alexa Fluor 488 (Invitrogen, 1:200) and Alexa Fluor 647 secondary antibodies (Invitrogen, 1:200). The sections were examined under a fluorescence microscope (BIOREVO BZ-9000; Keyence Corp, Osaka, Japan) after staining the cells with 0.5 μM TO-PRO-3 for 60 min.

### Morphological observation

Sections of the fifth lumbar (L5) spinal cord segment were sequentially immunostained with several antibodies using an immunofluorescence system. The fluorescent images were analyzed using a fluorescence microscope (BIOREVO BZ-9000; Keyence Corp, Osaka, Japan). Target antigen-positive and -negative motor neurons with diameters larger than 20 μm by TO-PRO-3 staining were separately counted in four sections per mouse. In this study, TO-PRO-3 was used as a cell marker throughout our experiments^28^,^46^,^47^. In addition, to analyze motor neurons more selectivity, ChAT-positive AHCs were counted in five sections immunostained using goat anti-ChAT polyclonal antibody (Milllipore Cat. No. AB144P) for each mouse. The number of AHCs in the ventral gray matter (ventral to the horizontal line running though the ventral edge of the central canal) was counted in five L5 sections for each mouse.

### Statistical analysis

Averaged data are presented as means and s.e.m. Statistical analysis was performed using JMP 12 software (SAS Institute, Inc.). For statistical comparisons of two groups, we used unpaired two-tailed Student’s t-tests. Differences were considered significant when P < 0.05.

## Additional Information

**How to cite this article**: Yamashita, T. *et al*. Calpain-dependent disruption of nucleo-cytoplasmic transport in ALS motor neurons. *Sci. Rep.* **7**, 39994; doi: 10.1038/srep39994 (2017).

**Publisher's note:** Springer Nature remains neutral with regard to jurisdictional claims in published maps and institutional affiliations.

## Supplementary Material

Supplementary Information
