# Supplementary material for: Calpain-dependent disruption of nucleo-cytoplasmic transport in ALS motor neurons
Source: Sci Rep. 2017 Jan 3;7:39994. doi: 10.1038/srep39994 (PMC5206745; doi:10.1038/srep39994)
Supplement: Supplementary Information [file srep39994-s1.pdf]

Supplementary information

Calpain-dependent disruption of nucleo-cytoplasmic transport in ALS motor neurons

Takenari Yamashita[1], Hitoshi Aizawa[2], Sayaka Teramoto[1], Megumi Akamatsu[1] and Shin Kwak[1, 3, *]

[1] *Graduate School of Medicine, The University of Tokyo, 7-3-1 Hongo, Bunkyo-ku, Tokyo 113-0033, Japan.*

[2] *Department of Neurology, Tokyo Medical University, 6-7-1 Nishishinjuku, Shinjuku-ku, Tokyo 160-0023, Japan.*

[3] *Clinical Research Center for Medicine, International University of Health and Welfare, 6-1-14 Konodai, Ichikawa, Chiba 272-0827, Japan.*

[*] *Correspondence should be addressed to S.K. (kwak-tky@umin.ac.jp).*

Supplementary Figures

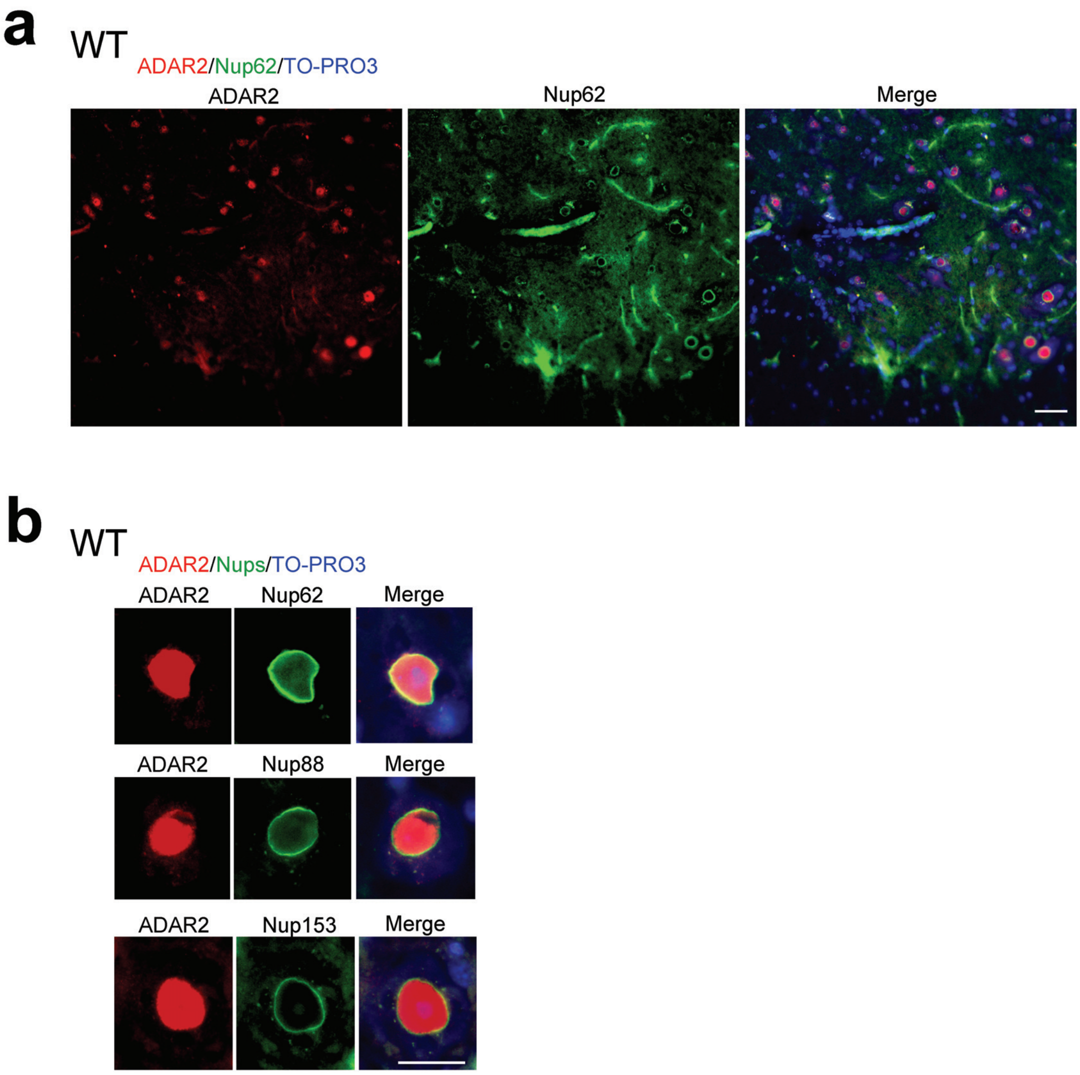

Supplementary Figure 1| Immunofluorescence staining for ADAR2 and Nup62 in the spinal cord of wild type mouse.

(**a**) Immunofluorescence for ADAR2 (red) and Nup62 (green) in the spinal cords of wild type mouse at 28 weeks of age. Scale bar, 50 μm. (**b**) In figure 1a, individual Immunofluorescence image of ADAR2 and Nup62, 88, and 153. TO-PRO-3 is a cellular staining marker. Scale bar, 20 μm.

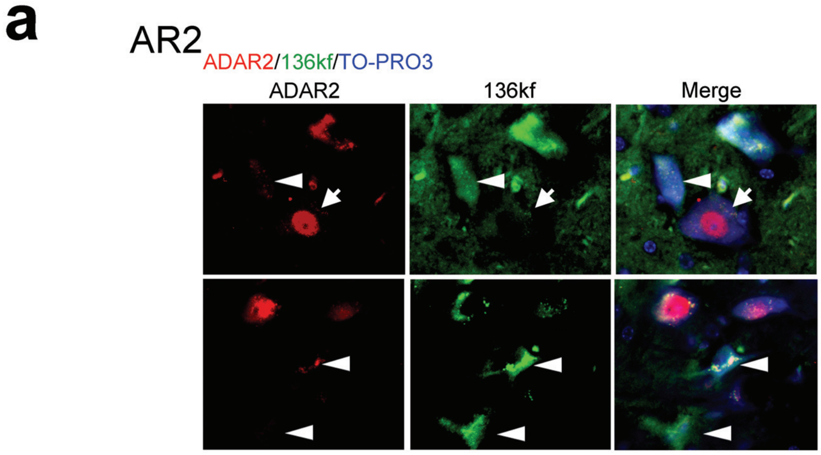

Supplementary Figure 2| Immunofluorescence staining for ADAR2 and 136kf in the spinal cord of AR2 mouse.

(**a**) Immunofluorescence for ADAR2 (red) and 136kf (green) in the spinal cords of AR2 mouse at 26 weeks of age. ADAR2 positive AHCs were not observed 136kf immunoreactivity (arrow). Immunoreactivity for 136kf was observed in AR2 mouse AHCs lacked ADAR2 expression (arrowheds). TO-PRO-3 is a cellular staining marker.

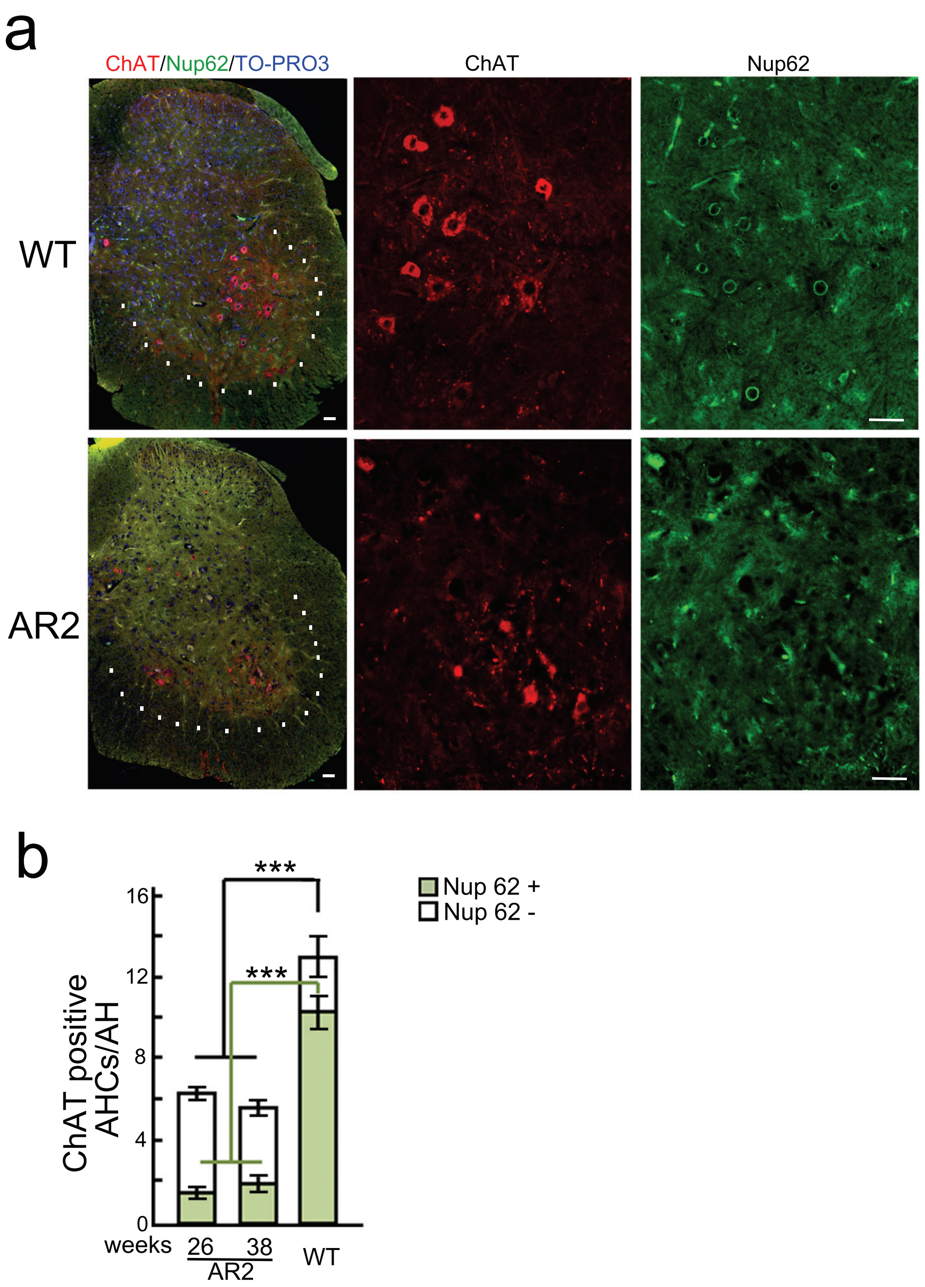

Supplementary Figure 3| Immunofluorescence staining for ChAT and Nup62 in the spinal cord of AR2 mice.

(**a**) choline acetytrasferase (ChAT; red) and Nup62 (green) immunostaining of the lumbar spinal cord. White dotted lines indicate the margin of the ventral gray matter. TO-PRO-3 (blue) was used as a cell marker[28,46,47]. The scale bar indicates 20 µm. (**b**) The number of ChAT and Nup62 double-positive AHCs was decreased in the AR2 mice compared to that in the wild type mice (WT). Means (columns) and s.e.m. (bars) are indicated (n = 5; *** $P < 0.001$, **Mann -Whitney** U test against the value of wild type mice). Green column is Nup62 positive AHCs, white column is Nup62 negative AHCs.

a

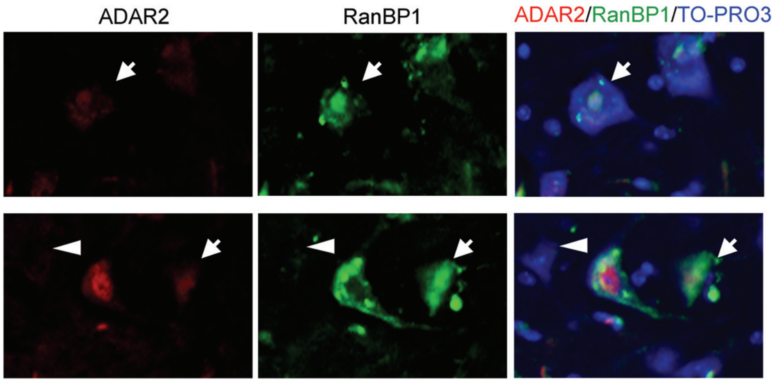

Supplementary Figure 4| Immunofluorescence staining for ADAR2 (red) and RanBP1 (green) in AHCs of a 12-week-old AR2 mouse.

(**a**) RanBP1 immunoreactivity was predominantly cytoplasmic in ADAR2-positive motor neurons. As the expression of ADAR2 decreased, RanBP1 immunoreactivity was increased in the nucleus and ultimately was specific to the nucleus (arrow). Moreover, RanBP1 immunoreactivity was depleted in ADAR2-lacking motor neurons (arrowhead).

a

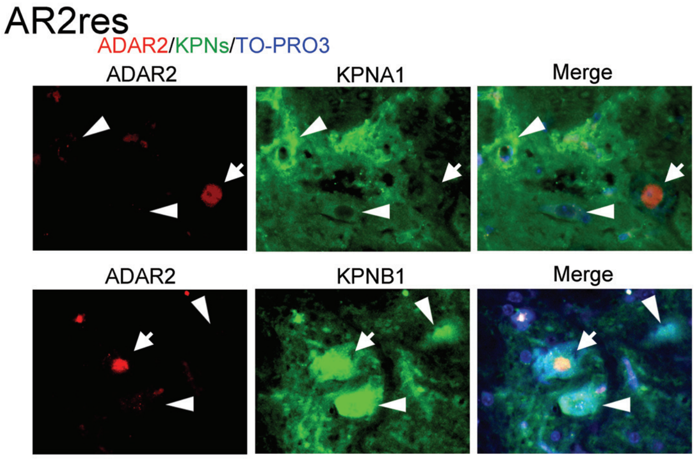

Supplementary Figure 5| Rescue of KPNA1 and KPNB1 in AHCs of AR2res mouse. Immunofluorescence for ADAR2 (red) and KPNA1 or KPNB1 (green) in the spinal cords of AR2res mouse at 28 weeks of age. There was normal KPNA1 and KPNB1 immunoreactivity in ADAR2-deficient AHCs (arrowheads) in the AR2res mouse. TO-PRO-3 is a cellular staining marker.

a

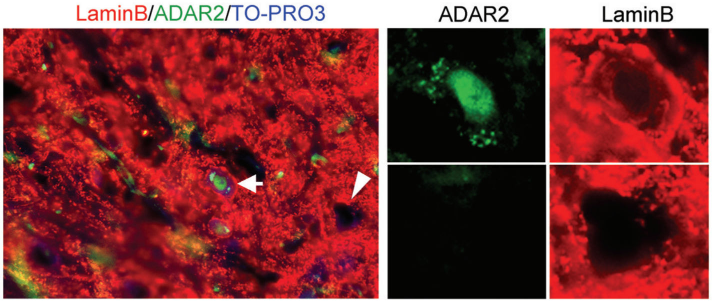

Supplementary Figure 6| Immunofluorescence staining for ADAR2 and lamin B in the spinal cord of AR2 mouse.

Immunofluorescence for ADAR2 (green) and lamin B (red) in the spinal cords of AR2 moues at 26 weeks of age. Cytoplasmic and perinuclear lamin B immunoreactivity was detected in ADAR2 positive AHCs (arrow) but not in ADAR2 negative AHCs (arrowhead) of AR2 mouse. TO-PRO-3 is a cellular staining marker.

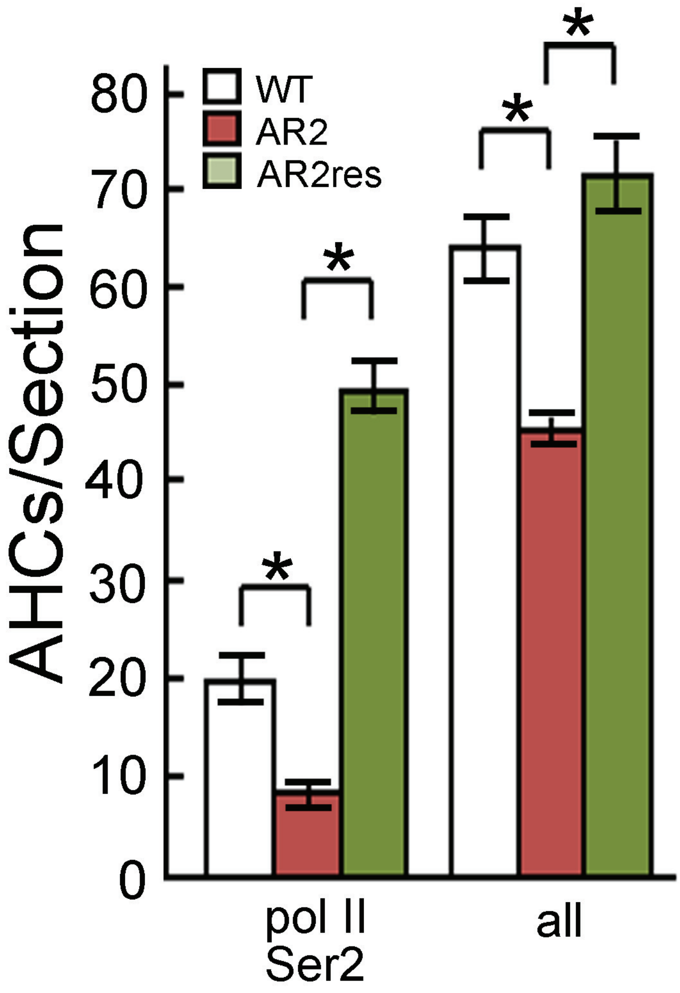

Supplementary Figure 7| The number of total and pol II Ser2-positive AHCs. Significantly fewer total and pol II Ser2-positive AHCs were observed in AR2 mice than in control wild type and AR2res mice. Means (columns) and s.e.m. (bars) are indicated (n = 5; *P < 0.05 compared to the cell counts in wild-type and AR2res mice, Student's t-test).

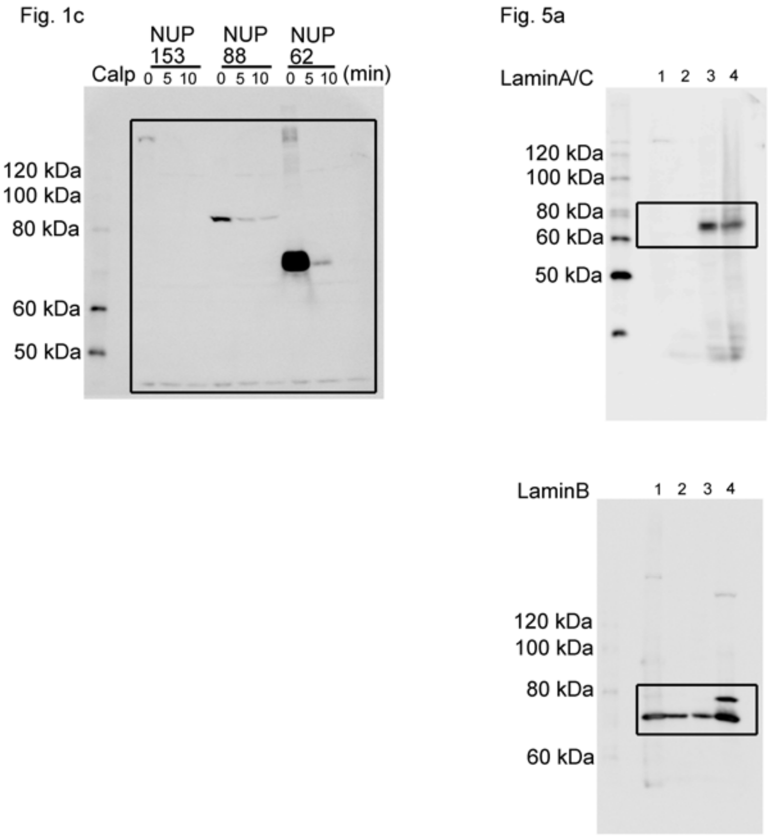

Supplementary Figure 8| Full-length pictures of the blots presented in main figures.
